# Body Mass Index and Poor Self-Rated Health in 49 Low-Income and Middle-Income Countries, By Sex, 2002–2004

**DOI:** 10.5888/pcd12.150070

**Published:** 2015-08-20

**Authors:** Aolin Wang, Onyebuchi A. Arah

**Affiliations:** Author Affiliation: Onyebuchi A. Arah, Department of Epidemiology, Fielding School of Public Health and Center for Health Policy Research, University of California, Los Angeles, Los Angeles, California; California Center for Population Research, Los Angeles, California.

## Abstract

This study investigated whether the relationship between body mass index (BMI) and poor self-rated health differed by sex in low-income countries and middle-income countries. We analyzed data from the World Health Survey (2002–2004) on 160,099 participants from 49 low-income and middle-income countries by using random-intercept multilevel logistic regressions. We found a *U*-shaped relationship between BMI and poor self-rated health among both sexes in both low-income and middle-income countries, but the relationship differed by sex in strength and direction between low-income countries and middle-income countries. Differential perception of body weight and general health might explain some of the observed sex differences.

## Objective

Excess weight, calculated as body mass index (BMI), is linked to poor self-rated health, a robust predictor of mortality and objective health (1,2). Findings are inconsistent on how this relationship differs by sex (3–5) in high-income countries, and empirical evidence is lacking in low-income and middle-income countries, where the prevalence of obesity is growing. Understanding the sex differences in the relationship between BMI and health is valuable for planning appropriate health promotion programs for each sex. Our study examined whether sex modifies the effect of BMI on poor self-rated health in low-income countries versus middle-income countries.

## Methods

We analyzed data from the cross-sectional World Health Survey (2002–2004) conducted by the World Health Organization in 70 countries (6). Only participants who were aged 18 years or older and had complete data on all variables were included in our analysis; our sample consisted of 160,099 participants from 22 low-income countries and 27 middle-income countries as defined by the World Bank in 2003 (7).

Self-rated general health was assessed by the question, “In general, how would you rate your health today?” The 5-point Likert scale response to this question was converted into a binary indicator comparing poor health (reported “poor” or “very poor”) with good health (reported “very good,” “good,” or “moderate”). BMI was derived according to self-reported height and weight (weight in kilograms divided by height in meters squared) and was classified into 5 categories (8): underweight (BMI < 18.5), normal weight (BMI, 18.5–24.9), overweight (BMI, 25.0–29.9), obese class I (BMI, 30.0–34.9), and obese class II and III, BMI ≥ 35.0). Confounding variables were age, sex, marital status (currently married or cohabitating versus not), urbanicity (living in an urban or semi-urban area versus rural area), educational attainment (no formal education, some primary school, primary school completed, secondary school completed, high school completed, or college and beyond), smoking status (current smoker or not), alcohol use (ever use of alcohol or not), and national gross domestic product per capita (in current US dollars).

After summarizing the characteristics of the participants by sex in low-income countries and middle-income countries, we examined associations between BMI and self-related health by using random-intercept multilevel logistic regressions to account for the multilevel structure of the World Health Survey data, in which individuals were clustered by country. We used SAS version 9.3 (SAS Institute Inc) for all analyses.

## Results

In low-income countries, women had lower levels of education, lower rates of smoking and alcohol use, and were more likely to have high BMI and report poor self-rated health than men (Table). In middle-income countries, educational attainment was similar for both sexes; women were less likely to live in urban areas, smoke, or drink alcohol but more likely to have high BMI and report poor self-rated health than men. Overall, participants from the middle-income countries, compared with participants from low-income countries, were slightly older, less likely to be married or cohabitating, and more likely to complete secondary school or beyond; a greater percentage in middle-income countries smoked and used alcohol.

**Table:** Sex-Specific Characteristics of 160,099 Participants From 49 Low-Income and Middle-Income Countries in World Health Survey, 2002–2004

Characteristic	Low-Income Countries (n = 22)		Middle-Income Countries (n = 27)	
	Men	Women	Men	Women
**Total, n**	31,437	32,478	43,358	52,826
**Age, mean (SD), y**	38.3 (15.4)	38.1 (15.7)	41.4 (16.2)	41.4 (16.3)
**Married or cohabitating, n (%)**	21,590 (68.7)	21,001 (64.7)	25,733 (59.4)	28,217 (53.4)
**Educational attainment, n (%)**				
No formal education	8,209 (26.1)	12,443 (38.3)	2,392 (5.5)	3,586 (6.8)
Some primary school	5,260 (16.7)	6,062 (18.7)	4,626 (10.7)	4,844 (9.2)
Primary school completed	7,119 (22.7)	6,479 (20.0)	9,750 (22.5)	10,858 (20.6)
Secondary school completed	5,160 (16.4)	3,586 (11.0)	13,906 (32.1)	17,149 (32.5)
High school completed	2,979 (9.5)	2,082 (6.4)	8,181 (18.9)	10,409 (19.7)
College and beyond	2,710 (8.6)	1,826 (5.6)	4,503 (10.4)	5,980 (11.3)
**Lives in urban or semi-urban area, n (%)**	20,869 (66.4)	21,549 (66.4)	17,475 (40.3)	19,155 (36.3)
**Current smoker, n (%)**	11,624 (37.0)	2,588 (8.0)	17,722 (40.9)	6,121 (11.6)
**Ever used alcohol, n (%)**	11,172 (35.5)	4,673 (14.4)	27,021 (62.3)	20,746 (39.3)
**BMI, n (%), kg/m^2^**				
Underweight (<18.5)	4,238 (13.5)	4,858 (15)	2,482 (5.7)	4,033 (7.6)
Normal (18.5–24.9)	20,562 (65.4)	19,402 (59.7)	23,701 (54.7)	26,952 (51.0)
Overweight (25.0–29.9)	4,465 (14.2)	5,235 (16.1)	12,959 (29.9)	14,131 (26.8)
Obese I (30.0–34.9)	812 (2.6)	1,312 (4.0)	3,079 (7.1)	5,356 (10.1)
Obese II and III (≥35.0)	1,360 (4.3)	1,671 (5.2)	1,137 (2.6)	2,354 (4.5)
**Poor or very poor self-rated health, n (%)**	2,041 (6.5)	2,984 (9.2)	2,695 (6.2)	4,599 (8.7)

Abbreviation: BMI, body mass index.

Overall, we found *U*-shaped associations between BMI and poor self-rated health among both sexes in low-income countries and middle-income countries (Figure). With the exception of the stronger association between being underweight and reporting poor self-rated health among men (odds ratio [OR] = 1.92; 95% confidence interval [CI], 1.69–2.17) than among women (OR = 1.42; 95% CI, 1.27–1.58), the strength of the associations between high BMI and poor self-rated health were similar in both sexes in low-income countries (*P* value for interaction = .03). However, associations between high BMI and poor self-rated health were slightly stronger among women than among men in middle-income countries (*P* value for interaction < .001). Being overweight was slightly negatively associated with poor self-rated health among men (OR, 0.86; 95% CI, 0.74–0.99) and women (OR = 0.78; 95% CI, 0.69–0.88) in low-income countries, but this association was reversed for women in middle-income countries (OR = 0.89, 95% CI, 0.80–0.98 [men]; OR = 1.12; 95% CI, 1.03–1.21 [women]).

**Figure:**
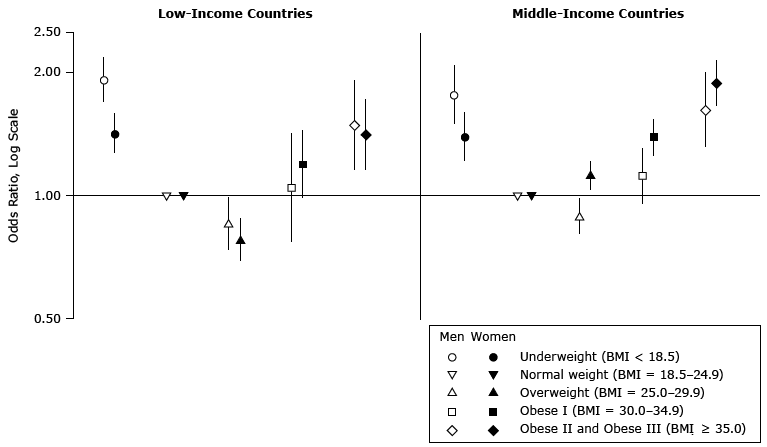
Sex-specific associations between body mass index (BMI) and poor self-rated health in 49 low- and middle-income countries. Data were obtained from multilevel multivariable adjusted regression analysis of the World Health Survey, 2002–2004 (N = 160,099). The model was adjusted for age, age-squared, marital status, urbanicity, educational attainment, smoking status, alcohol use, and national gross domestic product per capita. **Table:** 

Body Mass Index, kg/m^2^	Men (95% Confidence Interval)	Women (95% Confidence Interval)
**Low-income countries**		
Underweight (<18.5)	1.92 (1.69–2.17)	1.42 (1.27–1.58)
Normal (18.5–24.9)	1 (Reference)	1 (Reference)
Overweight (25.0–29.9)	0.86 (0.74–0.99)	0.78 (0.69–0.88)
Obese I (30.0–34.9)	1.05 (0.78–1.41)	1.20 (0.99–1.44)
Obese II and III (≥35.0)	1.49 (1.16–1.91)	1.41 (1.16–1.72)
**Middle-income countries**		
Underweight (<18.5)	1.76 (1.49–2.08)	1.39 (1.22–1.60)
Normal (18.5–24.9)	1 (Reference)	1 (Reference)
Overweight (25.0–29.9)	0.89 (0.80–0.98)	1.12 (1.03–1.21)
Obese I (30.0–34.9)	1.12 (0.95–1.31)	1.39 (1.25–1.54)
Obese II and III (≥35.0)	1.62 (1.32–1.99)	1.88 (1.65–2.14)

## Discussion

The *U*-shaped associations between BMI and poor self-rated health found in this study could indicate how overweight and underweight were perceived differently as countries underwent different stages of socioeconomic development. In undernourished areas, excess weight might be perceived as wealth or health, with underweight being associated with the wasting phase of diseases such as human immunodeficiency virus and acquired immune deficiency syndrome. On the other hand, the stigma of obesity is growing globally as obesity rates rise (9). In some of the more developed countries, obesity is increasingly considered a disease rather than a risk factor for chronic diseases.

The stigma of excess weight and the promotion of slim ideal body types through the mass media could drive the differences between the sexes in the relationship between BMI and poor self-rated health in middle-income countries. Women and men may differentially perceive their health according to their weight status. Women may be more likely than men to consider being overweight or obese a health problem (10), and overweight and obesity may constitute an important dimension of self-rated health for women (5). Overweight and obese women face greater societal pressures, such as weight discrimination and body image problems, than do men (11). Such differential perceptions and experiences of body weight and health may also contribute to documented differences between the sexes in self-related health and mortality (12).

Our study analyzed standardized global health data collected across the world and adjusted for both contextual and compositional confounding factors. However, the cross-sectional nature of the data limits our ability to draw causal inferences. Data on self-reported height and weight, commonly used in large-scale studies like ours, can induce BMI misclassification and attenuate associations with outcomes.

We found that sex modifies the associations between BMI and poor self-rated health differently in low-income countries versus middle-income countries. Such differences between the sexes could be considered when studying and acting on the determinants of health.
